# Editorial: Multipotent stromal cells and microenvironment of the tissue healing

**DOI:** 10.3389/fbioe.2023.1127703

**Published:** 2023-01-12

**Authors:** Ahmed Lotfy, Yvonne Reinwald, Jun Kobayashi, Jehan J. El-Jawhari

**Affiliations:** ^1^ Department of Surgery, Medical University of South Carolina, Charleston, SC, United States; ^2^ Department of Engineering, School of Science and Technology, Nottingham Trent University, Nottingham, United Kingdom; ^3^ Institute of Advanced Biomedical Engineering and Science, Tokyo Women’s Medical University, Tokyo, Japan; ^4^ Department of Biosciences, School of Science and Technology, Nottingham Trent University, Nottingham, United Kingdom; ^5^ Department of Clinical Pathology, Faculty of Medicine, Mansoura University, Mansoura, Egypt

**Keywords:** multipotent stromal cells, microenvironment, tissue healing, growth factors, biomaterials

Tissue regeneration is a vital process maintaining the survival and functions of different biological organs and systems. The regenerative process usually consists of three stages involving the body’s response following tissue damage due to trauma, pathological mechanisms, autoimmunity, or chemicals/toxins (Eming, Martin, and Tomic-Canic 2014). These regenerative phases include inflammation, repair, and remodelling. Multipotent stromal cells, also known as mesenchymal stem/stromal cells (MSCs), are known to play a role in all three stages of tissue regeneration. These stromal cells have multiple and unique functions compared to other eukaryotic cells. MSCs can differentiate into specific cells belonging to bone, cartilage, fat, skin, tendon, brain, and other tissue (Krampera et al., 2007; Lotfy et al., 2019). Additionally, these stromal cells can interact with various immune cells, thus modulating the innate and adaptive immune response. Besides differentiation and immunomodulation, MSCs have other support functions for hematopoietic stem cells helping blood cell regeneration (Fajardo-Orduña, 2015) and endothelial cells promoting angiogenesis (Tao et al., 2016).

The therapeutic potential of MSCs is highly considered, and extensive research is ongoing for their use in several degenerative conditions and diseases (Margiana et al., 2022). Some crucial components that supplement the regenerative capability of MSCs include biomaterials that provide 3D support for cell survival and functions, as well as growth factors as supplementary factors for MSCs-based therapies (Gu et al., 2021). It is essential to understand how native or transplanted stromal cells can interact with the components of the healing microenvironment. Such knowledge will enrich the understanding of the tissue regeneration process. Furthermore, research into the crosstalk between stromal cells and healing microenvironment components would have excellent value for identifying cellular, molecular, and biomaterial candidates that can promote stromal cell-based therapies.

Growth factors that support MSC healing are widely used as recombinant proteins, but these proteins have limitations of short half-life and off-target effects. Certelli et al. showed that fibrin hydrogel could help to deliver simultaneously two growth factors supporting angiogenesis. Co-delivery of recombinant VEGF and PDGF-BB aided angiogenesis and vascular formation in an experimental model of skin injury in mice. Another noted advantage of this growth factor co-delivery was the reduction of vascular hypertrophy that can be caused by the sole application of VEGF. Future investigations will be needed to modify biomaterials to control further the delivery of more than one growth factor in different degenerative conditions.

In osteoarthritis (OA), the inflammatory environment of the joint may affect the ability of MSCs to functionally integrate into the host tissue and exert beneficial effects, especially OA joint presents an altered level of inflammatory mediators. In this context, Marsh et al. studied in their research article the effects of leading interleukins IL-1β, IL-8 and IL-10 on MSCs *in vitro*, focusing on their impact on cell differentiation and the possible underlying mechanisms of action. They found that IL-1β, a pro-inflammatory mediator linked to OA pathophysiology, negatively affects MSC differentiation potential *in vitro* by reducing their capacity to form cartilage and significantly inducing osteogenesis.

MSCs sheet engineering is another example showing how the microenvironment affects MSC function. MSCs sheet engineering as a cell transplantation method allows MSCs to be collected in a sheet form while retaining their extracellular matrix by culturing them on a temperature-responsive culture dish. This method is being clinically used in different diseases, and its safety and efficacy have been confirmed. Kaibuchi et al. summarized in their review article the therapeutic effect of MSCs sheet in Medication-Related Osteonecrosis of the Jaw (MRONJ). In this review, the *in vitro* and animal studies of MSCs sheet applications in MRONJ as well as the future perspective and possible clinical trials, were discussed.

MSCs also are highly affected by any drugs or chemicals in the microenvironment. Acar et al. studied the effect of Metformin Senomorphics on prolonged *in vitro* cultured MSCs. They found that metformin reduced the senescence and cell death associated with prolonged *in vitro* cultivation. Moreover, they demonstrated that the senomorphic function of metformin is related to its reactive oxygen species (ROS) scavenging activity since in metformin-treated MSCs, the CEBPA, TP53 and USF1 transcription factors appeared to be involved in the regulation of several factors (SOD1, SOD2, CAT, GLRX, GSTP1) blocking ROS.

In addition to the classic use of reparatory cells, growth factors or biomaterials, targeting molecules linked to healing processes is increasingly explored for tissue regeneration. Cui et al. demonstrated that overexpression of miR-760 helped to increase collagen II and aggrecan *via* targeting the MyD88/NF-KB signalling pathway. These data support the value of targeting miRNA for enhancing intervertebral disc regeneration. In another work by Xu et al., osteoblast-derived exosomes were found to express high levels of miR-21. The same research group showed that miR-21 helps hematopoietic stem cell recovery following radiation damage, consequently inducing peripheral blood cell renewal.

We hope the novel discoveries and insights provided by these research and review articles will increase our understanding of the interaction between the MSCs and the microenvironment in tissue healing and develop and push MSC and related regenerative therapy forward to be a routine therapeutic tool in the near future.
